# Concerning FDA approval of trilaciclib (Cosela) in extensive-stage small-cell lung cancer

**DOI:** 10.1016/j.tranon.2021.101206

**Published:** 2021-08-19

**Authors:** Kerrington Powell, V Prasad

**Affiliations:** a College of Medicine, Texas A&M Health Science Center, College Station, TX 77843, United States; b Department of Epidemiology and Biostatistics, University of California San Francisco, 550 16th St, 2nd Fl, San Francisco, CA 94158, United States

## Abstract

Trilaciclib is a recently approved cyclin-dependent kinase 4/6 inhibitor that is designed to decrease the incidence of chemotherapy-induced myelosuppression in adult patients with extensive-stage small-cell lung cancer receiving chemotherapy. Currently, this first-in-class therapy raises two open issues: its bio-plausibility and paucity of evidence demonstrating a lasting impact on clinical endpoints. Based on the existing phase 2 data, trilaciclib appears to be a therapy that can make a positive impact by preventing myelosuppression, but empirical validation with larger phase III trials should be conducted to confirm these benefits. The purpose of this article is to facilitate discussion about the role of trilaciclib in clinical practice and the need for additional trials.

## Highlights

• Trilaciclib has a high level of bioplausibility, however further study is necessary.
• Many concerns remain about the evidence used to approve trilaciclib.
• There is some evidence that trilaciclib improves health-related quality of life.
• The small sample sizes may dampen the measurement of secondary outcomes.
• It is important to conduct well-powered phase III studies based on phase II results.

On February 12th, 2021, the FDA approved trilaciclib (Cosela, G1 Therapeutics), a cyclin-dependent kinase 4/6 inhibitor (CDK4/6), to lower the rate of chemotherapy-induced myelosuppression (CIM) in adult patients with extensive-stage small-cell lung cancer (ES-SCLC) receiving platinum/etoposide-containing or topotecan-containing regimens [1]. Here, we describe the data supporting marketing authorization and open questions regarding the evidence base.

## Trilaciclib to decrease chemotherapy-induced myelosuppression

Trilaciclib, the first-in-class therapy designed to reduce CIM's frequency, acts as a competitive inhibitor of CDK 4/6 to protect hematopoietic lineages from DNA damage of chemotherapy by reversibly inducing G1 cell cycle arrest. In CDK4/6-dependent cells (*e.g*., hematopoietic stem and progenitor cells (HSPCs) and lymphocytes), this arrest aims to facilitate myelopreservation and T lymphocyte activation, and preclinical data also suggest a potential increased susceptibility to immune checkpoint inhibitors (ICIs) [2].

Approval of this drug was based on three early randomized, double-blind, placebo-controlled studies [3], [4], [5]. Combined, these studies evaluated the effectiveness of the study treatment in only 240 patients. Although all of the trials met their primary endpoints, which included a decrease in the duration of severe neutropenia (DSN) and the rate of severe neutropenia (SN), it is unclear if these reductions will provide a meaningful benefit to patients [3], [4], [5]. While improvements in patient health-related quality of life (HRQoL) are a possible advantage of the new approval, not all research assessed HRQoL outcomes [4], and the extent the decreases in adverse events translates to meaningful outcomes will be addressed. Here, we identify two open issues raised by this first-in-line therapy: its bio-plausibility, lack of data showing an impact on clinical endpoints.

## Bioplausibility

First, ES-SCLC is a rapidly growing cancer. The proliferation of ES-SCLC contributes to its 8-to-13-month median survival and its susceptibility to chemotherapeutic agents [6]. The premise of utilizing a CDK4/6 inhibitor to minimize CIM is seemingly contradicted by the possibility that the same drug may result in chemotherapeutic antagonism. In other words, CDK4/6 inhibitors may shield both hematopoietic stem and cancer cells from the toxicity of chemotherapy. This problem is apparently resolved in the case of SCLC because of both SCLC's chemosensitivity and deficiency of retinoblastoma (*RB*) tumor suppressor protein, which theoretically minimize concerns of anti-tumor efficacy [7].

*RB* loss is associated with resistance to CDK4/6 inhibitors and presents in SCLC, making trilaciclib a rational therapeutic agent to minimize CIM toxicity in ES-SCLC patients. Researchers take one step further, providing a rationale that trilaciclib may even enhance chemotherapeutic benefit by minimizing dose delays, reductions, and immunodeficiency, thereby realizing the full use of chemotherapy and immunotherapies [8]. However, against this hypothesis, one must consider the aggressive nature of ES-SCLC and that *RB* inactivation among these tumors is *not* universal. Genomic profiles of 108 tumors from stage I-IV SCLC showed 93% *RB* inactivation [9], but with rapidly growing malignancies, anything less than 100% *RB* inactivation may undermine chemotherapy's effectiveness in some cells; thereby antagonizing chemotherapy efficacy.

Furthermore, it is also possible that reducing the chemotherapeutic dose will have a similar effect to trilaciclib. This hypothesis is reinforced by the increase in chemotherapeutic dose reductions in the placebo arm of the Weiss et al. study (35.1% vs. 7.9%); however, there were no significant differences in overall survival or response rates [4]. If trilaciclib is antagonistic to chemotherapy regimens, it is reasonable to assume that these studies may be comparing antagonistic treatment to full-dose chemotherapy. This begs the question: what studies are needed to tease apart these hypotheses?

Second, the randomized studies' sample sizes are too small to rule in or rule out a meaningful interaction or potential detriment of the therapy in this disease setting. Researchers confront this limitation, stating several possible reasons why there was no significant improvement in anti-tumor efficacy from the use of trilaciclib [3]. However, there are limitations that the researchers do not address, such as the restriction of administering prophylactic granulocyte colony-stimulating factors (G-CSFs) in cycle (C) 1 in all of the studies, even though DSN in C1 was evaluated as the primary endpoint in these trials [3], [4], [5]. Depending on the study, the average reduction of DSN in C1 was either four days or five days, which are statistically significant differences but may not be clinically meaningful [3], [4], [5]. The problem is worsened in the Hart et al. trial since prophylactic G-CSF administration is the standard of care given the prevalence of FN with topotecan-containing regimens [5]. It is plausible that administration of GCSF in C1 could result in the same time reduction, and because there are no differences in patients for RBC transfusion or G-CSF administration in either the Daniel et al. or Hart et al. trials, one may even argue that patients are being switched from RBC transfusions to intravenous administration of a separate costly medication [3,5]. Furthermore, the small sample sizes may dampen the measurement of secondary outcomes, such as febrile neutropenia or antibiotic usage, which may diminish the unveiling of potential harms (e.g., worse overall survival). Regardless of the disease's pathophysiology, a phase III trial alone can illuminate a potential survival decrement or antagonized anti-tumor efficacy.

Finally, our analysis forces us to face the question: why was trilaciclib approved based on phase II data alone? The OS results from the phase II studies should prompt researchers to conduct adequately powered phase III trials to reveal a potential signal (Table). We also know phase II trials–studies performed to determine if a drug continues to have promise or activity (i.e., hypothesis-generating)–may not predict phase III results [10]. Additionally, in a highly lethal cancer like ES-SCLC–a randomized phase III trial would neither be difficult nor time-consuming to show survival outcomes [11]. Although the decrease in AEs and marginal gains in HRQoL seem to be a step forward, there are still concerns about whether trilaciclib truly improves outcomes. For example, in the Daniel et al. trial, there was no significant difference in patient-reported outcomes (PROs) assessing HRQoL [3]. And even though the trial by Hart and colleagues demonstrated a marginal improvement, the benefit may be driven by a statistical phenomenon since the placebo group had a higher HRQoL than the interventional arm at baseline [5]. In other words, the placebo group may have deteriorated more rapidly, a phenomenon called regression towards the mean. That said, in the absence of definitive randomized data, counseling patients will remain difficult until phase III trials provide data from which conclusions might be drawn. At $34,000 per treatment, physicians and patients should not accept the uncertainty of anti-tumor efficacy, quality of life, and survival outcomes in a novel therapy [12].

**Table:** Overall survival results in early phase randomized trials of trilaciclib.

Study	Median OS for Trilaciclib	Median OS for Placebo	Hazard Ratio & Confidence Interval
Study 1: Trilaciclib prior to etoposide, carboplatin, and tezolizumab (E/P/A) [3]	12.0 (9.6, 16.2) months	12.8 (7.9, 15.5) months	HR (95% CI): 0.92 (0.57, 1.49) *P* = 0.8228
Study 2: Trilaciclib prior to etoposide and carboplatin (E/P) [4]	10.9 (9.1, 16.4) months	10.6 (7.7, 15.2) months	HR (80% CI): 0.87 (0.61, 1.24) *P* = 0.6107
Study 3: Trilaciclib prior to topotecan [5]	6.2 months	6.5 months	HR (80% CI): 1.36 (0.96, 2.01) *P* = 0.3377

Based on the current phase II data, trilaciclib appears to be an intervention that may make a positive impact by preventing CIM, maintaining immune system function, and minimizing cytotoxic AEs, although many of these outcomes remain unclear. Using this drug may augment chemotherapy and ICI regimens, but whether or not trilaciclib helps patients live longer or live better is a matter of continuing debate and uncertainty. Trilaciclib has elements of bioplausibility, but molecular determinants of CDK4/6-independence and dependence are complex, and the therapy must prove itself with empirical validation. Phase III trials testing survival, anti-tumor efficacy, and quality of life outcomes are the only way forward. By doing so, we can safely implement this therapy to provide a clinical benefit to patients.

## CRediT authorship contribution statement

**Kerrington Powell:** Data curation, Writing – review & editing, Writing – original draft. **V Prasad:** Conceptualization, Data curation, Writing – review & editing.

## Declaration of Competing Interest

Disclosure: Vinay Prasad's Disclosures. (Research funding) Arnold Ventures (Royalties) Johns Hopkins Press, Medscape, MedPage (Consulting) UnitedHealthcare. (Speaking fees) Evicore. New Century Health (Other) Plenary Session podcast has Patreon backers. All other authors have no financial nor non-financial conflicts of interest to report.

## References

[1] Commissioner of the FDA approves drug to reduce bone marrow suppression caused by chemotherapy. U.S. Food and Drug Administration. https://www.fda.gov/news-events/press-announcements/fda-approves-drug-reduce-bone-marrow-suppression-caused-chemotherapy. Published February 12, 2021.

[2] Lai A.Y., Sorrentino J.A., Dragnev K.H. (2020). CDK4/6 inhibition enhances anti-tumor efficacy of chemotherapy and immune checkpoint inhibitor combinations in preclinical models and enhances T-cell activation in patients with SCLC receiving chemotherapy. J. Immunother. Cancer.

[3] Daniel D., Kuchava V., Bondarenko I. (2020). Trilaciclib prior to chemotherapy and atezolizumab in patients with newly diagnosed extensive-stage small cell lung cancer: a multicentre, randomised, double-blind, placebo-controlled phase II trial [published online ahead of print, 2020 Dec 21. Int. J. Cancer.

[4] Weiss J.M., Csoszi T., Maglakelidze M. (2019). Myelopreservation with the CDK4/6 inhibitor trilaciclib in patients with small-cell lung cancer receiving first-line chemotherapy: a phase Ib/randomized phase II trial. Ann. Oncol..

[5] Hart L.L., Ferrarotto R., Andric Z.G. (2021). Myelopreservation with trilaciclib in patients receiving topotecan for small cell lung cancer: results from a randomized, double-blind, placebo-controlled phase II study. Adv. Ther..

[6] Demedts I.K., Vermaelen K.Y., van Meerbeeck J.P. (2010). Treatment of extensive-stage small cell lung carcinoma: current status and future prospects. Eur. Respir. J..

[7] Roberts P.J., Kumarasamy V., Witkiewicz A.K., Knudsen E.S. (2020). Chemotherapy and CDK4/6 Inhibitors: unexpected bedfellows. Mol. Cancer Ther..

[8] Mackall C.L. (1999). T-cell immunodeficiency following cytotoxic antineoplastic therapy: a review. Oncologist.

[9] George J., Lim J., Jang S. (2015). Comprehensive genomic profiles of small cell lung cancer. Nature.

[10] (2019). Olaratumab for STS disappoints in phase III. Cancer Discov..

[11] Chen E.Y., Joshi S.K., Tran A., Prasad V. (2019). Estimation of study time reduction using surrogate end points rather than overall survival in oncology clinical trials. JAMA Intern. Med..

[12] FDA Approves (2021). Cosela: anton Rx report. Anton Health.

